# Process performance and microbial community dynamics in semi-dry mesophilic co-digestion of crop residues and livestock manure

**DOI:** 10.1371/journal.pone.0345783

**Published:** 2026-04-09

**Authors:** Xiaoli Zhu, Guowei Chen, Jia Liu, Yanping Xu, Abdullah Gera, Lina Luo, Santao Chou

**Affiliations:** 1 Weifang Institute of Technology, Weifang, P. R. China; 2 Weifang Commercial College Shandong Province, Weifang, P. R. China; 3 Dezhou Weili Engineering Environmental Protection Technology Co., Ltd, Dezhou, P. R. China; 4 College of Engineering, Northeast Agriculture University, Harbin, P. R. China; Tsinghua University, CHINA

## Abstract

Semi-dry anaerobic co-digestion represents a promising strategy for efficient resource utilization of diverse agricultural wastes; however, its operational performance under mesophilic conditions and the underlying microbial mechanisms remain insufficiently understood. In this study, a 35-day semi-dry anaerobic co-digestion experiment was conducted using corn stover, wheat straw, cattle manure, and chicken manure as co-substrates, under 12% total solids and 37 °C, to elucidate biogas product dynamics and microbial community succession. The methane production exhibited a distinct biphasic pattern, marked by an initial decline, a prolonged lag phase, and a single, sharp peak that culminated between days 21 and 23. The peak methanogenic potential estimated using the modified Gompertz model reached 137 mL·g^-1^ VS, indicating incomplete substrate degradation within 35 days. Acetic acid, total volatile fatty acids, and chemical oxygen demand all reached their maximum values on day 15, reflecting intensive hydrolysis and acidogenesis during the first 15 days. In contrast, the lowest pH value was observed on day 15, while ammonia nitrogen concentration fluctuates between 3.5 and 4.0 mg/L. Microbial diversity analysis showed higher bacterial diversity on day 20, and increased archaeal diversity on day 30. The dominant bacterial phyla included Firmicutes, Bacteroidota, Proteobacteria, Actinobacteriota and Fusobacteriota with *Caldicoprobacter*, *Fastidiosipila*, *Tissierella*, and *Alkaliphilus* identified as the most abundant genera, playing key roles in organic matter degradation and acetate metabolism. Among archaea, Crenarchaeota was the predominant phylum, followed by Euryarchaeota and Korarchaeota. In summary, the semi-dry co-digestion system of the four agricultural wastes was characterized by the dominance of hydrogenotrophic methanogens (*Methanobrevibacter*) coupled with the syntrophic acetate oxidation pathway under conditions of high ammonia nitrogen. These findings provide mechanistic insights into microbial regulation of semi-dry multi-substrate anaerobic digestion and support its potential application in agricultural waste management.

## Introduction

In China, the annual production of corn stalk and wheat straw amounts to several billion tons as a result of widespread corn and wheat cultivation. When effectively utilized as renewable energy resources, these residues can be transformed from potential environmental burdens into valuable assets [1]. In addition, the annual generation of livestock and poultry manure in China has exceeded 4 billion tons, representing another abundant, cost-effective and sustainable source of renewable energy [2]. Among the various direct and indirect pathways for biomass utilization, anaerobic digestion stands out as an efficient and environmentally sound approach for converting complex biomass to biogas through the coordinated activity of microbial communities under anaerobic conditions [3].

Anaerobic digestion can process a wide range of organic materials and may be categorized according to specific operational conditions, such as temperature and total solids (TS) content [4]. Temperature is a critical parameter because of its strong influence on microbial community composition, which governs the degradation of organic matter. Room temperature, mesophilic (30–40℃) and thermophilic (50–60 ℃) conditions have all been reported as feasible; however, mesophilic temperature is generally considered most suitable for the digestion of agro-industrial wastes [5]. In addition, mesophilic anaerobic digestion systems typically require low operation and maintenance costs [6]. Another crucial factor for stable and efficient operation is the TS content. Although wet anaerobic digestion is well-established, its requirement for high water input poses significant economic and logistical challenges when treating high TS substrates such as agricultural residues. Operating at higher TS levels allows for increased organic loading and results in the generation of smaller volumes of biodigested slurry (BS), which is produced in large quantities annually and often represents a bottleneck for the routine operation of biogas plants. However, excessive organic loading can lead to early digester failure [2]. Consequently, semi-dry anaerobic digestion systems with TS contents between 10% and 20% may represent a more sustainable option for digester plants, as they reduce water consumption compared with low TS systems (0 < TS ≤ 10%), while avoiding the operational risks associated with high TS systems (20% ≤ TS ≤ 40%).

In addition, substrate composition plays a decisive role in determining biogas production performance during anaerobic digestion. Generally, feedstocks with high concentrations of readily degradable organic matter (e.g., volatile solids, VS) generate substantially greater biogas production [7]. Anaerobic digestion based on a single substrate often fails to accommodate the compositional complexity of agricultural wastes and may also limit the economic viability of the anaerobic digestion system. For instance, biogas production is typically low when cattle manure is used as the sole feedstock in cattle farming operations [8]. Consequently, the co-digestion of livestock manure with crop residues has attracted increasing interest from researchers. In an anaerobic digestion system co-treating cattle manure and wheat straw, no significant differences in biogas yield were observed; however, the volumetric biogas production increased to over 1.11 L_biogas_/L_reactor_ compared with mono-digestion of cattle manure [8].

In China#39;s intensive livestock regions, the co-digestion of cow manure and chicken manure is widely practiced in biogas plants, whereas the incorporation of wheat straw and corn stalks remains limited. Although these crop residues are produced in large quantities in rural areas, their utilization in anaerobic digestion systems is constrained by handling and processing difficulties. Moreover, most previous studies on anaerobic co-digestion have focused on binary substrate combinations (e.g., manure with crop straw) under wet or dry conditions. Consequently, a significant knowledge gap exists regarding the systematic optimization, process stability, and underlying microbial mechanisms of co-digestion systems that involve three or more complex organic wastes under semi-dry conditions. Under conditions of high TS content and multi-substrate composition often accompanied by elevated ammonia nitrogen concentrations, the associated fermentation performance and microbial responses remain poorly understood. This lack of knowledge limits the practical application of anaerobic digestion technology for integrated organic waste management.

Therefore, this study aims to address this gap by: (1) systematically evaluating the methane production performance and process stability of a multi-substrate combination comprising cow manure, chicken manure, wheat straw and corn stalk under mesophilic conditions; (2) elucidating the microbial community dynamics and metabolic pathways that drive the observed synergies. We believe this work provides novel insights into designing efficient and robust multi-feedstock anaerobic digestion systems.

## Materials and methods

No specific permits were required for the described study because: (1) the anaerobic sludge used as inoculum was obtained from Shandong Luxi Dasheng Environmental Protection Technology Co., LTD with the company’s administrative approval for research use; (2) fermentation materials were collected from privately owned and managed farms with the owner’s consent, and the materials are not subject to any conservation regulations; (3) the laboratory experiments did not involve endangered or protected species.

### Experimental materials

Wheat straw and corn stalks were collected from the local agricultural area in Weifang, Shandong Province. Following natural air and sun drying, they were pulverized using an FW135 (Beijing Ever Bright Medical Treatment Instrument Co., Ltd., Beijing, China) and sieved through a 40-mesh screen. Cow and chicken manure were obtained from Yuquanwa Ecological Sightseeing Park in Weifang, Shandong Province. The BS used as the inoculum for this experiment was from a stably operating anaerobic digester at Shandong Luxi Dasheng Environmental Protection Technology Co., LTD, which utilized local chicken and cow manure as primary raw materials. Pre-incubation of the inoculum was performed under anaerobic conditions at 37 ± 0.2°C for one to two weeks to minimize endogenous gas production. The characteristics of both the fermentation inoculum and substrates are detailed in Table 1.

**Table 1:** Raw material and inoculum characteristic parameters.

Item	TS/%	VS (Wet Basis)/%	Total Organic Carbon content/%	Total nitrogen content/%
Wheat straw	89.8	82.6	44.5	0.446
Corn stalk	91.4	82.2	44.3	0.601
Cow manure	15.7	13.3	40.5	1.57
Chicken manure	39.1	30.5	38.1	4.22
Biodigested slurry	5.67	3.68	1.57	1.67

### Anaerobic digestion equipment

Methane production was automatically recorded using a MultiTalent 203 (Nova Skantek Instruments (Beijing) Co., Ltd, Beijing, China). This fully automatic methane potential tester included an anaerobic digestion unit, an acidic gas adsorption module, and a gas flow rate and data acquisition module. The anaerobic digestion unit was composed of an electrically-heated thermostatic water bath, fifteen 500 mL glass bottles and a matching mechanical agitator. The acidic gas adsorption module consisted of fifteen 100 mL glass bottles, each containing 80 mL 3 mol/L NaOH solution to absorb acidic gases. The equipment was used at room temperature.

### Experiment design

The bulk volume of raw anaerobic digestion material in each bottle was 350 g and the anaerobic digestion process lasted 35 days. The mixture was prepared at a dry mass ratio of 1.6 (chicken manure): 1 (cow manure): 1 (wheat straw): 1 (corn stalks) with a total carbon to nitrogen ratio of 20.2. The TS was adjusted to about 12%, using BS rather than deionized water. This strategy was adopted to simulate the digestate recirculation practice commonly applied in continuous industrial anaerobic digestion plants while simultaneously supplying moisture and an active microbial inoculum. The VS ratio of substrate to inoculum was maintained at 3.12. The same amount of BS was put into the bottles as a negative control (BS). After sufficient mixing of the substrates and BS in the glass bottles, which were positioned in the electric-heated thermostatic water bath at 37℃ ± 0.2℃. The mixture was intermittently stirred at 80 rpm, with 30-second mixing intervals every 3 minutes.

### Sample collection and measurements

During the 35 days of the anaerobic digestion process, samples were collected every 5-day intervals. Samples of negative control were collected on day 35. Three replicates were tested. The pH was measured by PHS-3E (INASE Scientific Instrument Co., Ltd., Shanghai, China) immediately after terminating the digestion by placing the bottle in an ice box. Aliquots of the fermentation liquid from each bottle were rapidly frozen in liquid nitrogen (−196°C) for 60 seconds, and subsequently were kept in a −80°C ultra-low temperature freezer for microbial community analysis. The fermentation liquid supernatant was collected by high-speed centrifugation (20,000 × g, 15 min, 4℃) and stored until the analysis of ammonia nitrogen, volatile fatty acid (VFA) and chemical oxygen demand (COD). The TS was determined by oven-drying at 105°C for 24h (WGL 45B, Tianjin Taisite Instrument Co., Ltd., Tianjin, China). Then the dried samples were ashed in a chamber furnace (SX-4–10, Tianjin Taisite Instrument Co., Ltd., Tianjin, China) at 550°C for 5 h to determine VS content by mass difference. The total carbon and nitrogen were analyzed using a Vario MAX cube (Elementar Analysensysteme GmbH, Langenselbold, Germany). VFA concentrations were analyzed according to Sun et al. [9]. Ammonia nitrogen concentration and COD content were assessed using Nessler#39;s reagent spectrophotometry method [10] and the fast digestion-spectrophotometric method [11], respectively.

Extraction of the genomic DNA from the fermentation liquid was conducted by E.Z.N.A.® Soil DNA Kit (Omega Bio-tek, Inc., Norcross, GA, USA). DNA purity and quantification were assessed on NanoDrop2000 (Thermo Scientific, Waltham, MA, USA). DNA integrity was verified via 1% agarose gel electrophoresis. After obtaining high-quality DNA, next-generation sequencing of 16S rRNA was carried out by Shanghai Biozeron Biotechnology Co., Ltd (Shanghai, China). The selected primers for 16S rRNA were: bacteria 341F (5’-CCTAYGGGRBGCASCAG-3’) and 806R (5’-GGACTACHVGGGTWTCTAAT-3’); archaea Arch519F (5’-CAGCCGCCGCGGTAA-3’) and Arch915R (5’-GTGCTCCCCCGCCAATTCCT-3’). PCR amplification products were electrophoresed through 2% agarose gels and purified using the AxyPrep DNA Gel Extraction Kit (Axygen Scientific Inc., San Diego, CA, USA). Quantifications of purified PCR products were performed using Qubit^®^3.0 (Invitrogen, Carlsbad, CA, USA). Sequencing was performed using NovaSeq PE250 (Illumina, Kapa Biosciences, Woburn, MA, USA) after library construction by the NEXTFlex Rapid DNA-Seq Kit (Bioo Scientific, Austin, TX, USA). Taxonomic classification was performed using the SILVA database (Release 138.1; available at: http://www.arb-silva.de).

### Dynamics

The methane production data were fitted by the modified Gompertz equation, as P = Pm × exp {-exp [Rm × e × (*λ- t*) /Pm + 1]}, where P = cumulative methane production (mL·g^-1^ VS); Pm = theoretical maximum methane yield (mL·g^-1^ VS); Rm = peak methanogenic rate (mL·g^-1^ VS d^-1^); t = anaerobic digestion time (d); λ = methanogenesis lag phase (d), and e = base of natural logarithm (2.7183). The non-linear curve fitting was performed using the NLFit tool in OriginPro 2021 (OriginLab Corporation, USA) and the goodness of fit was assessed by the R² value.

### Statistics

Statistical analysis was performed using SPSS Statistics 22 software (IBM, Armonk, NY, USA). GraphPad Prism 6 (GraphPad Software, San Diego, CA, USA) and Origin 2021 (OriginLab, Northampton, MA, USA) were used for graphs. The Weighted_Unifrac algorithm was employed for principal coordinate analysis (PCoA), using sequence abundance data categorized at the operational taxonomic unit (OTU) level to identify clustering patterns among the study subjects.

## Results

### Daily and cumulative methane production

**Fig 1:**
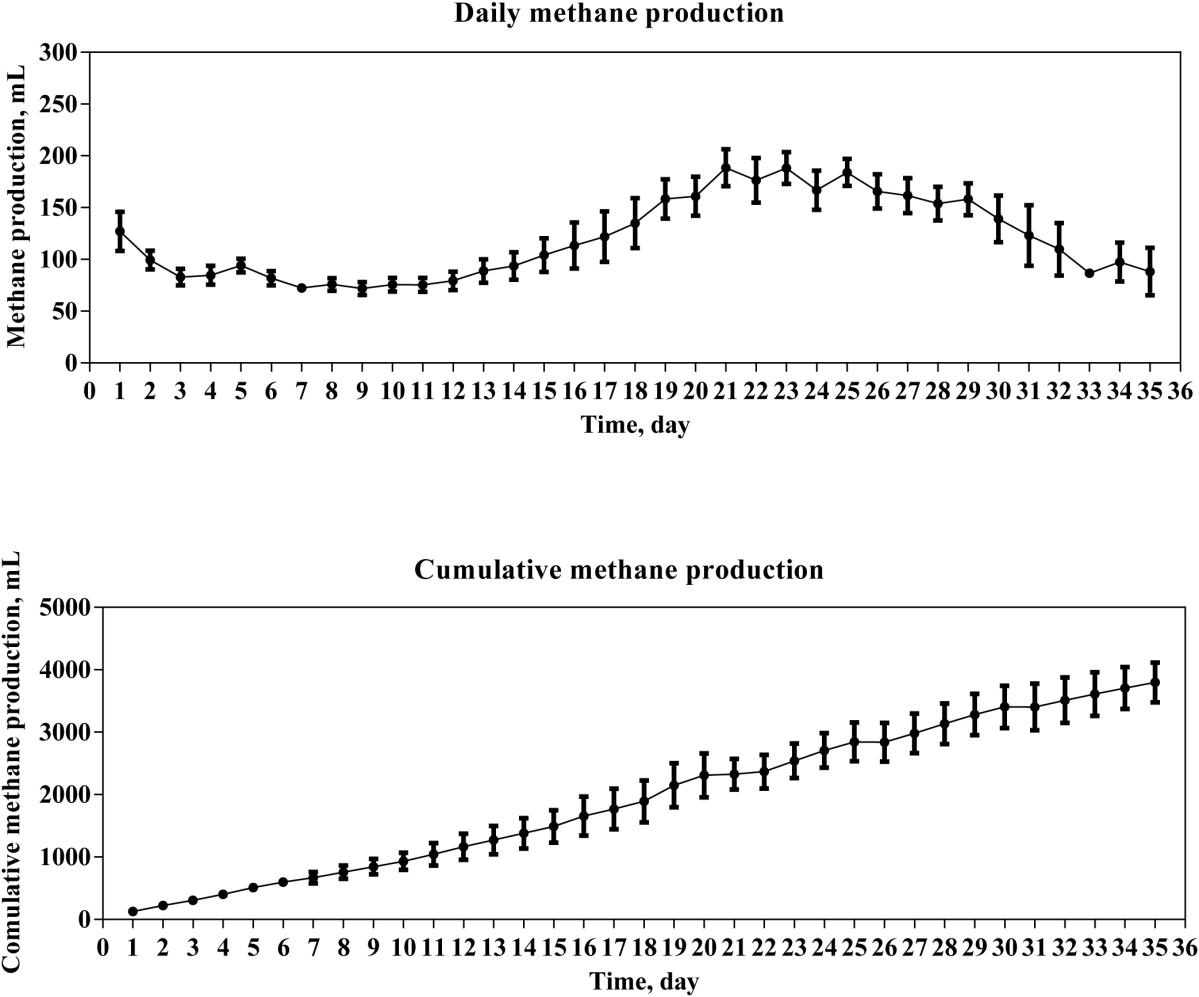
Daily methane production and Cumulative methane production.

**Fig 2:**
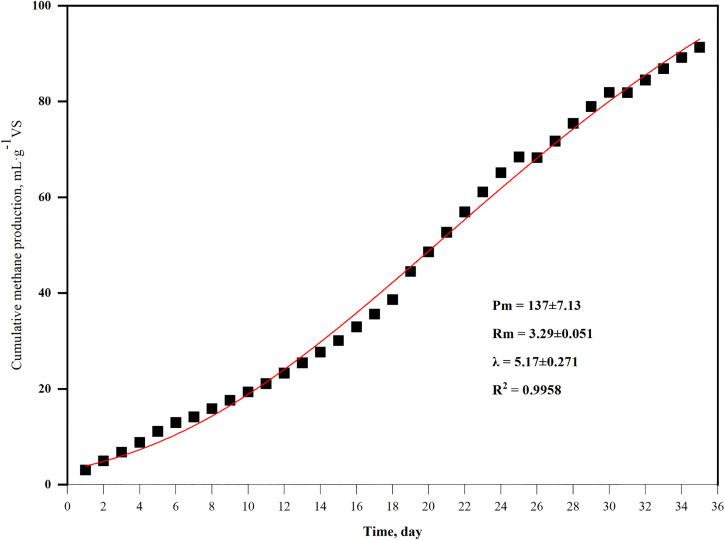
Cumulative methane production characteristics of moderate temperature anaerobic fermentation of agricultural residues of the modified Gompertz model.

Daily methane production was initially high, reaching 127 mL, and then gradually decreased to 71.9 mL on day 9 (Fig 1). From day 9 to day 21, daily methane production rose slowly, reaching a maximum daily methane production of 188 mL on days 21 and 23, and afterwards, it exhibited an overall downward trend. Cumulative methane yield increased steadily over the 35 days, ultimately reaching 3796 mL. Fig 2 summarizes the kinetic parameters obtained from the model fitting. The high R²value (0.9958) indicates that the modified Gompertz model effectively described the methane production kinetics. The estimated peak methanogenic potential was 137 mL·g^-1^ VS. The maximum methanogenic rate was 3.29 mL/g VS per day and the retention time was 5.17 d. Blank bottles containing only BS produced no measurable biogas, confirming that there was no endogenous gas production from the inoculum itself under the experimental conditions.

### Volatile fatty acids production, pH value, COD, ammonia nitrogen content, TS and VS

**Fig 3:**
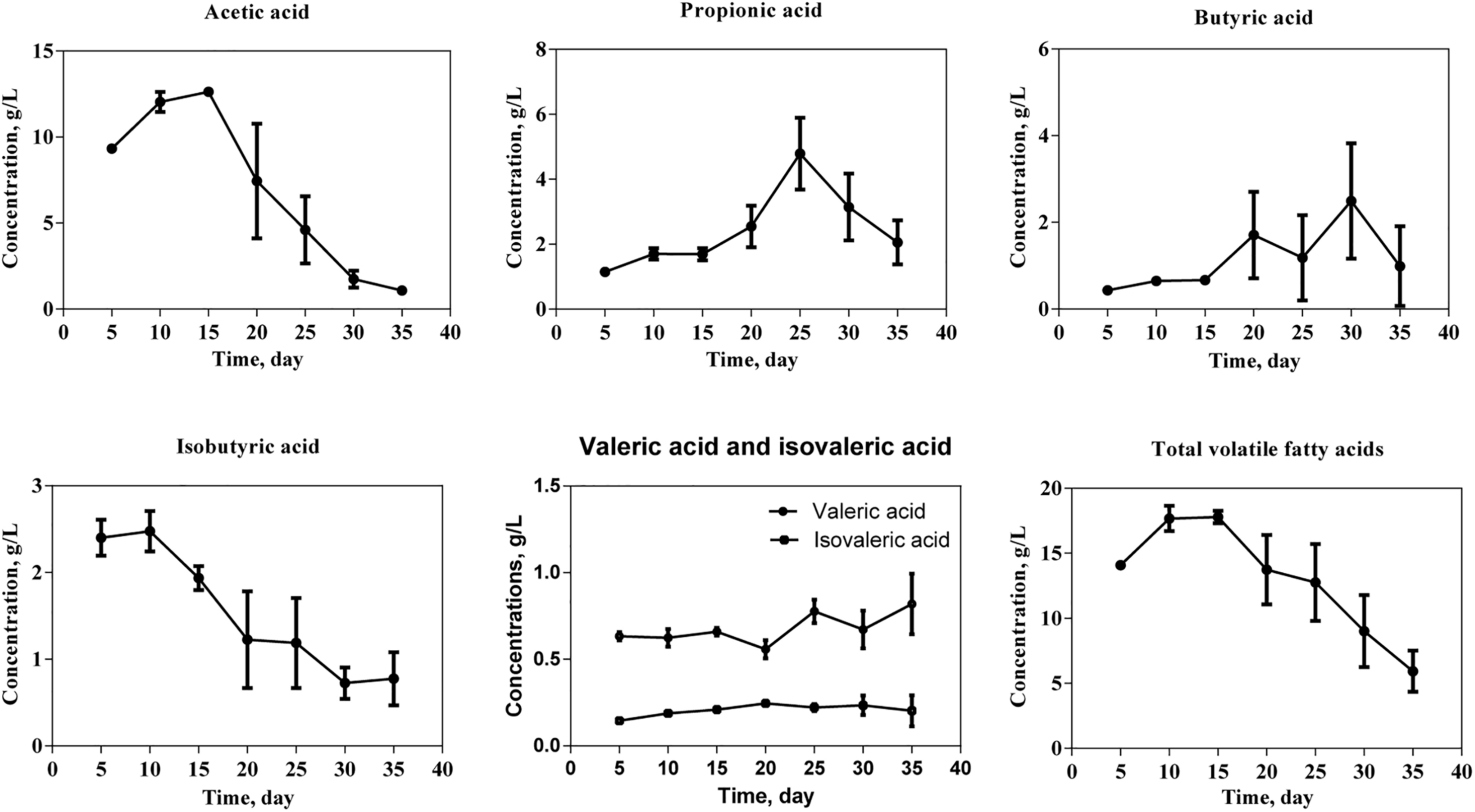
Volatile fatty acids production of moderate temperature anaerobic fermentation of agricultural residues.

The acetic acid concentration increases gradually during the initial fermentation phase, peaks at 15 days, and subsequently declines (Fig 3). The highest time spot of the propionic acid concentration was day 25, followed by a gradual decrease. Butyric acid concentration exhibited fluctuating but overall slightly increasing trends during the fermentation period, whereas isobutyric acid presented a progressive fall after day 10. Low variability throughout fermentation was observed on valeric and isovaleric acids. The total VFA concentration peaked around day 15 during the 35-day fermentation period and then decreased gradually.

**Fig 4:**
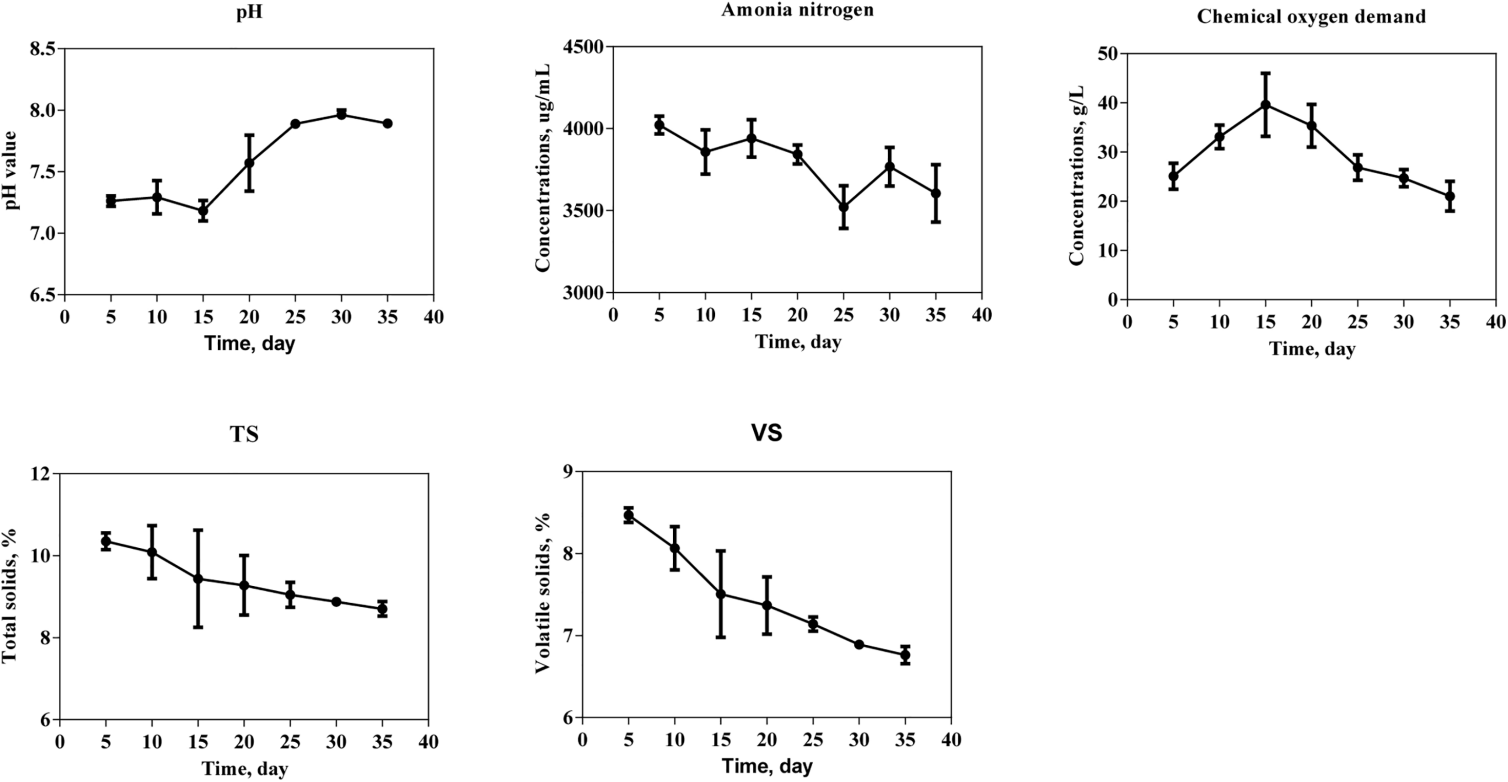
Physicochemical indexes of moderate temperature anaerobic fermentation of agricultural residues.

An initial decrease in pH was observed, reaching a minimum value of 7.18 on day 15, followed by a gradual increase to a peak of 7.96 by day 30 (Fig 4). Ammonia nitrogen concentration appeared to show a wavy downward trend between days 5 and 35, reaching its lowest level (3,522 µg/mL) on day 25. COD reached its maximum value of 39.6 g/L on day 15 and then decreased progressively, resulting in lower concentrations on day 35 compared to those on day 5. As the digestion proceeded, the TS contents decreased from an initial 12% to 8.70% while VS (wet basis) contents declined to 6.76% on day 35.

### Microbial diversity

**Fig 5:**
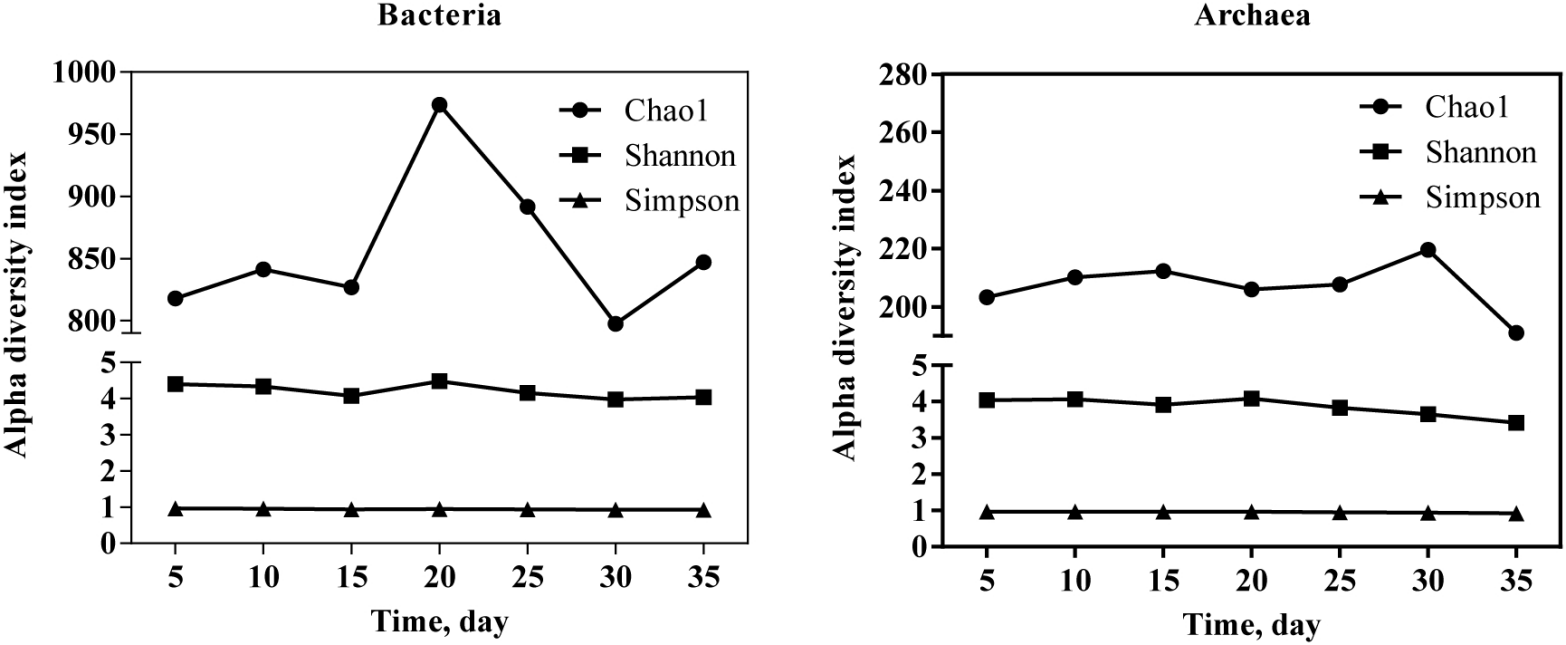
Microbial alpha diversity analysis of moderate temperature anaerobic fermentation of agricultural residues.

After pretreatment, the analysis yielded 1333 bacterial OTUs and 1008 archaeal OTUs from all samples. Among alpha diversity indexes (Fig 5), the Chao 1 index was highly variable, with the highest Chao 1 values for bacterial communities on day 20 and for archaeal communities on day 30. In contrast, the lowest Chao1values of bacteria and archaea were observed on days 30 and 35, respectively. On day 20, the largest Shannon indices for both bacterial and archaeal communities were observed as 4.48 and 4.09, respectively. The Simpson index exhibited relatively minor variation throughout the digestion process for both microbial domains, ranging from 0.929 to 0.964 for bacteria and from 0.926 to 0.971 for archaea. Specifically, the Simpson index for bacteria ranged from 0.929 to 0.964, while that for archaea ranged from 0.926 to 0.971.

**Fig 6:**
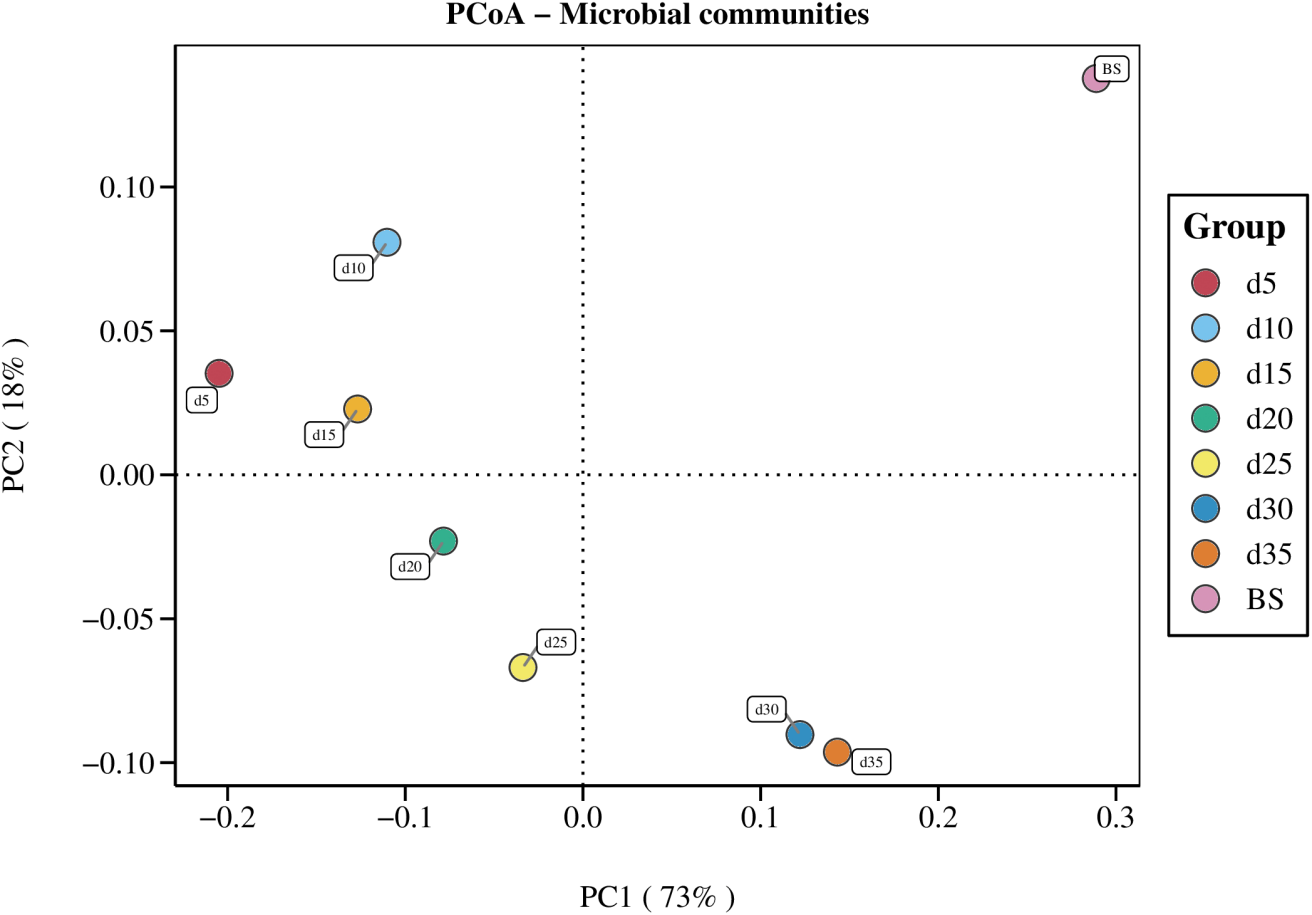
Principal coordinate analysis (PCoA) of fermentation samples collected in different time spots based on Weighted_Unifrac distances. PC1 explained 73% of the variation observed, and PC2 explained 18% of the variation. BS means Biodigested slurry.

PCoA based on OTU abundance showed the variation of microbiota compositions in the fermentation system over time (Fig 6). The samples from different time points were dispersed and gradually shifted along the PC1 axis from day 5 to day 35. The BS sample was separated from the others.

### Microbial community structure

#### Bacterial community structure.

**Fig 7:**
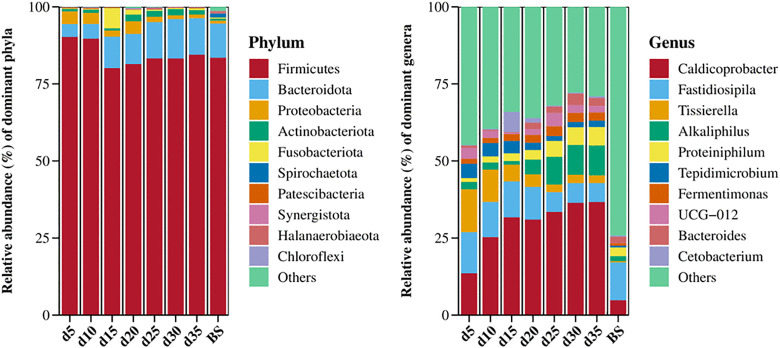
Relative abundance of bacterial community at the phylum and genus levels. BS means biodigested slurry.

Select the top 10 phyla and genera based on abundance for display, and classify other species as “Others” (Fig 7). At the phylum level of bacteria, Firmicutes, Bacteroidota, Proteobacteria, Actinobacteriota, and Fusobacteriota were the foremost five dominant phyla. The relative abundance of Firmicutes was higher before day 10 but fell thereafter, while Bacteroidota showed an opposite performance. The relative abundance of Proteobacteria decreased after day 20. The relative abundance of Actinobacteriota remained below 1% before day 15, but increased to above 1% at later stages. The relative abundance of Fusobacteriota reached its highest level (6.53%) on day 15. The bacterial community in the BS group was dominated by Firmicutes and Bacteroidota, with all other detected phyla exhibiting relative abundances below 2%.

Among all bacterial genera, *Caldicoprobacter* exhibited the highest relative abundance during the anaerobic digestion process. It was followed by *Fastidiosipila*, *Tissierella*, *Alkaliphilus*, *Proteiniphilum*, *Tepidimicrobium*, *UCG-012*, *Fermentimonas*, *Bacteroides* and *Jeotgalibaca*. *Caldicoprobacter*, *Alkaliphilus* and *Proteiniphilum* increased as fermentation progressed; on the contrary, *Fastidiosipila*, *Tissierella* and *Tepidimicrobium* diminished in the later stage. In the BS group, these ten genera collectively constituted 25.8% of the relative abundance; this group was dominated by *Fastidiosipila* (12.3%), followed by *Caldicoprobacter*, *Proteiniphilum*, *Bacteroides*, and *Alkaliphilus*.

#### Archaea community structure.

**Fig 8:**
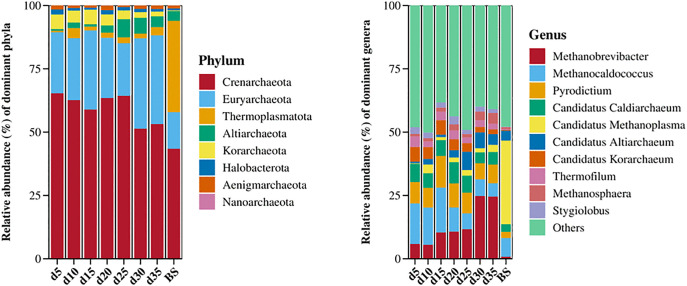
Relative abundance of archaeal community at the phylum and genus levels. BS means Biodigested slurry.

A total of eight archaeal phyla were identified and the top 10 abundant genera are shown in Fig 8. Phylum-level analysis revealed that the most abundant phylum was Crenarchaeota, followed by Euryarchaeota, Korarchaeota, Altiarchaeota, Thermoplasmatota, Halobacterota, Aenigmarchaeota and Nanoarchaeota during the fermentation period. The relative abundance of Crenarchaeota exhibited a fluctuating pattern, with an initial decline, followed by a gradual increase after day 15 and a subsequent decrease after day 25, yet remaining consistently above 50%. Between days 5 and 25, the relative abundance of Euryarchaeota reached its peak on day 15. A general decline was observed in the relative abundance of Korarchaeota. The relative abundance of Altiarchaeota was greater in the period after day 25 compared to the period before it. Despite exhibiting fluctuations, the relative abundance of Thermoplasmatota did not exceed 4% at any time point. In the BS group, the relative abundance of Crenarchaeota was below 50%. Notably, the abundance of Thermoplasmatota was 36.0% of the population, substantially higher than that in the experimental group. Conversely, the relative abundance of Euryarchaeota in the BS group was lower than that in the experimental group.

At the genus level, *Methanobrevibacter* and *Methanocaldococcus* were relatively most abundant genera in the digestion system, followed by *Pyrodictium*, *Candidatus Caldiarchaeum*, *Candidatus Methanoplasm*, *Candidatus Altiarchaeum*, *Candidatus Korarchaeum*, *Thermofilum*, *Methanosphaera*, *Stygiolobus*. The relative abundance of *Methanobrevibacter* increased overall, whereas that of *Methanocaldococcus* showed the opposite trend. The peak relative abundance of *Pyrodictium* was observed on day 15. The relative abundances of the other genera showed dynamic fluctuations throughout the process. In the BS group, *Candidatus Methanoplasm* was the dominant genus with a relative abundance significantly higher than that of the experimental group, while the abundances of *Pyrodictium* and *Methanobrevibacter* were lower.

## Discussion

### Methane production

In an anaerobic co-digestion of pig manure and hulless barley straw lasting for 17 days, the methane volume showed two peaks, with the first peak at day 3 and the second peak at day 8 [12]. When the substrates are a mixture of pig manure, food waste and corn stover, two peaks are also observed on day 1 and day 6, respectively [13]. In the present study, a biphasic peak pattern was observed. The initial, small peak likely resulted from the rapid consumption of residual soluble substrates or trace oxygen by facultative microorganisms during startup. The utilization of BS introduced hydrolytic and methanogenic populations into the substrate mixture, potentially facilitating process initiation and enhancing early stability. This initial phase was followed by an extended lag period, during which strict anaerobic conditions were established and the hydrolysis of complex substrates proceeded more slowly. This observed decline in methane production can be attributed to a combination of factors, including the depletion of readily degradable substrates, inhibitory effects caused by VFAs accumulation and/or elevated ammonia nitrogen levels (Figs 3 and 4) and the time required for syntrophic microbial consortia to become functionally established. Ultimately, the enrichment and dominance of key hydrolytic and methanogenic communities on day 15 led to the main methane production peak around days 21–23, a conclusion strongly supported by VFA profiles and microbial community analyses (Figs 3, 7, and 8). The time difference in peak appearance may be attributed to the heterogeneous composition of the anaerobic fermentation substrate, which requires the collaboration of multiple microorganisms to complete hydrolysis and acidification. Moreover, the time it takes for the hydrolytic acid-producing microbial community to decompose different organic substances varies a lot [13]. Although the cumulative methane yield of 91.3 mL g ⁻ ¹ VS obtained in this study was relatively lower than that reported in co-digestion systems using two or three substrates [12–13], this result should be interpreted within the specific context of the feedstock characteristics employed in the present work. The lignocellulose-rich straws and nitrogen-rich manures used in the present study are individually known for their structural recalcitrance and a high risk of causing ammonia inhibition, respectively. Crucially, combining multiple substrates creates a more complex nutrient structure and microbial ecosystem, which can inherently challenge methanogenic efficiency and limit the ultimate yield [14]. Therefore, within this challenging framework, the observed methane yield represents a competitive outcome of multi-substrate co-digestion coupled with BS recirculation. Building on these findings, further enhancement in methane production may be achieved by targeted intervention strategies, such as the addition of specific trace elements [15].

### Physicochemical indexes

The minimum observed pH on day 15 coincided precisely with the peak acetic acid concentration, demonstrating its decisive influence on system acidity. The pH exhibited an initial decline followed by gradual recovery, a characteristic feature of stable anaerobic digestion, with all measured values remaining within the optimal neutral range (7.1–8.0) [16]. A similar phenomenon was reported in an anaerobic digestion system co-digesting food waste, pig manure, and corn stover at a ratio of 1:1:1 [13], highlighting the crucial role of balanced substrate composition in maintaining intrinsic buffering capacity. During the 35-day fermentation period, the total VFA concentration closely follows the dynamic pattern of acetic acid, peaking around day 15 before gradually declining, representing the hydrolysis and acidogenesis phases between day 0 and day 15. This correlation highlights acetic acid’s dominant contribution to the total VFA pool. The peak in acetic acid concentration on day 15 marks the transition from dominant acidogenesis to the onset of methanogenesis. Thereafter, VFAs were metabolized by microorganisms such as *Proteiniphilum* and *Tepidimicrobium*, generating acetate, hydrogen, and carbon dioxide, which were subsequently utilized by methanogens for methane production. The accumulated acetate thus served as the primary precursor for the subsequent methane production peak observed several days later (Fig 1), illustrating the inherent kinetic lag between intermediate formation and its final conversion. Conversely, the sustained presence and eventual decline of propionic acid after day 25 reflect a more complex degradation pathway. Its removal is a hallmark of efficient syntrophic metabolism, requiring the cooperative activity of syntrophic propionate-oxidizing bacteria and hydrogenotrophic methanogens. The successful depletion of propionate during the later digestion stage indicates that these essential syntrophic partnerships were effectively established, thereby contributing to overall process stability.

The COD serves as a measure of the system#39;s total potential energy, in that it quantifies the pool of organic matter accessible to methanogens as substrate. The COD reached its maximum level on day 15. It corresponds to the peak hydrolysis period, where a substantial amount of particulate organic matter (e.g., cellulose, hemicellulose) is solubilized, releasing a high concentration of intermediate compounds (e.g., soluble sugars and oligomers) into the aqueous phase. These compounds are subsequently converted to VFAs, as evidenced by the concurrent peak in acetic acid (Fig 3). As fermentation proceeded, these intermediates were subsequently utilized by methanogens, leading to the observed reduction in COD. Therefore, this COD peak was not an indicator of process inhibition but rather a dynamic reflection of intensive intermediate production prior to its final conversion to methane.

The lowest ammonia nitrogen concentration on day 25 was still higher than 3000 µg/mL, a level commonly associated with ammonia inhibition [17]. Combined higher ammonia nitrogen with lower methane yield compared with Han et al. [12] and Liu et al. [13] indicated that ammonia accumulation could exert an inhibitory effect on the methanogenic process [18,19], especially during the initial stage. Although elevated ammonia levels may have contributed to the early metabolic lag, the persistence of methane production throughout the process indicates that inhibition was effectively mitigated. The maintenance of a neutral pH favored the predominance of less toxic ammonium (NH₄⁺) form, and crucially, the microbial community shifted towards ammonia-tolerant, hydrogenotrophic methanogenesis pathway. This ecological adaptation highlights the system#39;s capacity to maintain functional stability under conditions of moderate ammonia stress.

Previous studies have shown that high TS content can negatively affect cumulative methane production by limiting substrate nutrient mass transfer and constraining microbial activity [16]. In the present study, both TS and VS contents declined gradually as the digestion proceeded. The net reduction of TS by one-third indicates the effective activity of the hydrolytic bacterial consortium. However, TS levels remained above 8% after 35 days of digestion, demonstrating that organic matter was not fully degraded and that residual substrates remained available for further methane production. This incomplete degradation partially explains the lower specific methane yield observed on a VS basis. Moreover, the use of recycled BS for moisture adjustment reduced the net water demand of the process, thereby addressing a key limitation commonly associated with wet anaerobic digestion systems.

### Microbial community succession

The microbial community structure underwent significant temporal shifts during the digestion process, with PC1 accounting for 73% of the variance. This result indicates a strong temporal gradient in community composition. Key environmental factors such as pH, metabolic byproducts, and substrate availability likely contributed to this dynamic. Discrepancies in the Chao1 indices of bacteria and archaea suggest distinct diversity patterns in the bacterial and archaeal populations during the anaerobic digestion process. The largest Shannon indices of both bacterial and archaeal communities were observed on day 20, indicating that species richness and evenness were highest at this stage. However, the Simpson index values remained consistently above 0.93, signifying that the community was dominated by a few highly prevalent species. Furthermore, the distinct separation of the BS sample from the others suggests that the initial inoculum community differed substantially from the stabilized communities established during the active fermentation process.

The gradual succession of the microbial community structure is intrinsically linked to functional shifts within the fermentation system. At the bacterial phylum level, Firmicutes, Bacteroidota, and Proteobacteria were the most prevalent and functionally significant groups in anaerobic fermentation, consistent with previous findings in mesophilic anaerobic digestion [12]. Within Firmicutes, numerous syntrophic species mediate the degradation of complex substrates into VFAs, for example, acetic acid, serving as the primary substrate for subsequent acetoclastic methane formation [20]. Therefore, it was the hydrolysis and acetic acid accumulation period before day 15, followed by acetoclastic methane production. Members of the phylum Bacteroidetes have been shown to produce diverse lytic enzymes (e.g., hydrolases, lipases) that play critical roles in breaking down complex organic substrates [12]. Similarly, species within Bacteroidetes and Proteobacteria demonstrate metabolic capacities involving degradation of complex organics coupled with assimilation of simple carbohydrates (e.g., glucose) as well as VFA [16]. Cloacimonetes are the fourth phylum in anaerobic digestion sludge [16], while in a trial of anaerobic co-digestion of pig manure and hulless barley straw, Synergistetes are the fourth phylum [12], different from Actinobacteriota in our findings. Correspondingly, Actinobacteriota is the fourth dominant phylum in an anaerobic digestion experiment of dairy sludge and sewage [21]. The Actinobacteriota phylum comprises metabolically versatile species capable of decomposing diverse substrates (carbohydrates, proteins) and hydrolyzing recalcitrant lignocellulose through extracellular enzyme secretion (cellulases, xylanases, peroxidases) [22], which played an eminent role in straw fermentation. Additionally, as reported by Shanahan et al. [23], Fusobacteriota possess specialized metabolic pathways for complex organic matter decomposition into acetic acid, suggesting their functional importance during the mid-phase degradation (days 10–15) of the anaerobic digestion processes.

At the genus level of bacteria, *Caldicoprobacter* species possess the ability to deconstruct plant biomass polysaccharides, such as cellulose and hemicellulose [24]. Similar to *Caldicoprobacter*, members of the genus *Alkaliphilus*, anaerobic alkaliphiles, are capable of degrading peptides and proteins [25,26]. *Proteiniphilum* under Bacteroidota serves as a key player in protein and acetate degradation and exhibits remarkable ammonia tolerance [27]. *Fastidiosipila* and *Tissierella* are proteolytic bacteria capable of utilizing amino acids to produce VFA [28,29]. *Tepidimicrobium species* have been found to exhibit high lignocellulolytic and cellulolytic activities and utilize propionate as a substrate rather than acetate [30]. Therefore, bacteria from the genera of *Caldicoprobacter*, *Alkaliphilus* and *Tepidimicrobium* contributed significantly to the degradation of the crude fiber in corn stover and wheat straw throughout the fermentation period, with the genus *Caldicoprobacter* being the main population. Regarding protein hydrolysis and utilization, *Fastidiosipila* and *Tissierella* dominated the initial to mid-fermentation period, whereas *Alkaliphilus* and *Proteiniphilum* predominated during the middle and late phases. Together, these genera contributed to organic matter breakdown and supplied critical metabolic intermediates to support methanogenesis.

Members of *Crenarchaeota* are obligate anaerobes with sulfur-dependent and ammonia-oxidising metabolisms, which could reduce NH_4_^+^ concentration, thereby mitigating the inhibition of methanogens [31,32]. *Methanobrevibacter* and *Methanocaldococcus*, both belonging to the phylum Euryarchaeota, were relatively abundant genera but exhibited opposite variable trends. *Methanobrevibacter* has been identified as the predominant methanogen in ruminants [33], likely originating from cow manure used in the present study. Over time, *Methanobrevibacter* became the most dominant component of the methanogen populations, replacing *Methanocaldococcus*, which typically grows with hydrogen or formate instead of complex organic substances [34]. This shift likely enhanced community resilience against ammonia inhibition and facilitated the oxidation of recalcitrant intermediates. It was observed that the methane production gradually increased as acetic acid was consumed. However, only one acetotrophic methanogen, *Methanosarcina*, which directly utilizes acetate for methanogenesis, was detected, and its abundance remained low (< 0.2). *Methanosarcina* species are metabolically flexible, capable of both acetoclastic and hydrogenotrophic methanogenesis, and they are tolerant to total ammonia nitrogen levels up to 7000 mg L ⁻ ¹ [6]. Given the low abundance of direct acetate-utilizers, we hypothesize that an efficient syntrophic cooperation existed between syntrophic hydrogen-producing acetogens and hydrogenotrophic methanogenic archaea. Specifically, the syntrophic acetate-oxidizing pathway exhibits a more competitive advantage over the direct acetate cleavage pathway. This phenomenon may be attributed to the inhibitory effect of high ammonia nitrogen concentration on *Methanosaeta*, another specialist acetate-degrader, which was not detected in the system [6]. In comparison, syntrophic hydrogen-producing acetogens and hydrogenotrophic methanogenic archaea possess stronger tolerance to ammonia nitrogen [35], enabling them to maintain their metabolic activities and drive methanogenesis efficiently under such environmental conditions. Furthermore, *Tepidanaerobacter*, belonging to Firmicutes, is capable of performing syntrophic acetate oxidation to hydrogen [34], and reached an average relative abundance of 0.570% from day 20 to day 35. *Methanobrevibacter* consumes hydrogen and maintains a low hydrogen partial pressure through methanogenesis. This indicated that the syntrophic acetate oxidation pathway was active in the system. *Tepidanaerobacter* and hydrogenotrophic methanogens formed an efficient and delicate syntrophic alliance, which jointly undertook the important task of degrading acetate and producing methane, thereby ensuring the stability of the anaerobic fermentation system.

As a hyperthermophilic methanogen, *Methanocaldococcus* may come from the substrate or the BS, but whether it remained metabolically active throughout the process requires further investigation. The relative abundances of *Pyrodictium* under Euryarchaeota and *Candidatus Caldiarchaeum* under Caldarchaeota didn’t fluctuate significantly across different time points, probably because they are autotrophic by using sulfur or hydrogen and can’t produce methane [36,37]. *Candidatus Korarchaeum*, under Korarchaeota, decreased, while *Candidatus Altiarchaeum*, under Altiarchaeota, increased as the fermentation progressed, living in symbiosis with methanogens [38,39]. Additionally, *Thermofilum* under Crenarchaeota, *Methanosphaera* under Euryarchaeota, *Stygiolobus* under Crenarchaeota and *Candidatus Methanoplasma* under Euryarchaeota were also identified, and the total relative abundance of these genera exhibits low population variability throughout the study.

It is important to note that bacterial and archaeal communities were assessed using separate analytical approaches. Therefore, this study does not present a direct quantitative comparison of their absolute abundances. Instead, our interpretation focuses on the relative temporal trends within each domain to infer functional shifts. The comparatively high abundance of well-recognized acidogenic bacterial phyla during the acid-accumulation phase (Fig 7), followed by the increased prominence of methanogenic archaea during the gas production (Fig 8), clearly demonstrates a successional transition in process dominance from acidogenesis to methanogenesis.

## Conclusion

This study demonstrates that semi-dry co-digestion of corn stalk, wheat straw, cow manure, and chicken manure under mesophilic conditions achieved a cumulative methane yield of 3796 mL with a peak methanogenic potential of 137 mL·g ⁻ ¹ VS. More importantly, the results revealed the microbial succession governing system performance characterized by an initial phase dominated by hydrolytic and acidogenic bacteria (e.g., *Caldicoprobacter*, *Alkaliphilus* and *Proteiniphilum*) responsible for substrate breakdown and VFA accumulation, followed by a critical transition toward hydrogenotrophic methanogens, primarily *Methanobrevibacter*, that drove the observed methane production peak. These findings establish a mechanistic link between specific microbial taxa (e.g., Fusobacteriota associated with acetogenesis) and process kinetics (VFA dynamics and methane generation), thereby advancing the fundamental understanding of multi-substrate anaerobic digestion. From an applied perspective, the identified feedstock formulation and insights into microbial consortia dynamics provide a practical reference for biogas plants seeking to co-process similar agricultural and livestock residues, with the potential to enhance methane yield and operational stability. The dominance of hydrogenotrophic methanogenesis further suggests that process optimization strategies (e.g., targeted pH regulation and trace element supplementation) could be implemented to favor this pathway and improve system efficiency. Overall, the successful management of this complex substrate mixture in a semi-dry digestion system offers a promising model for integrated organic waste treatment, contributing to both renewable energy production and local environmental remediation.
